# News and Notes

**Published:** 1901-08

**Authors:** 


### NEWS AND NOTES.

---

THE *Atlanta Medical and Surgical Journal* and *Southern Medical Record* having been consolidated into the ATLANTA JOURNAL-RECORD OF MEDICINE, exchanges will please be sent only to the latter. We are getting many duplicates.

---

VACCINATION has been made compulsory in Cuba.

---

SINCE July 1 patent medicines are no longer taxed.

---

DR. FLOYD W. MCRAE has sailed for Europe, expecting to be absent several months.

---

DR. DUNBAR ROY who was recently operated upon for appendicitis is now convalescent.

---

IN Baltimore 451 persons died the week ending July 6, of which 78 were from sunstroke.

---

DR. WILLIAM H. DALY, of Pittsburg, who committed suicide recently, was worth $250,000.

---

MR. FREDERICK TREVES has had conferred on him the honor of knighthood by the King of England.

---

BY the use of fireworks and explosives on the Fourth of July, 19 persons were killed and 1,611 were injured.

---

IN May 1,674 deaths from tuberculosis occurred in Colorado. In 224 cases the disease originated in Colorado.

BROOKLYN physicians are being sued by a debt-collecting agency, whose contracts they signed through misunderstanding.

---

N. S. DAVIS, JR., has been elected dean of the Northwestern University Medical School, and A. R. Edwards, secretary.

---

NEW MEXICO will have a medical examination for school-teachers. No person having tuberculosis will be allowed to teach.

---

A NEW YORK midwife operated on a tongue-tied child, severing an artery from which the child bled to death. She will be tried for manslaughter.

---

IN Indianapolis it is alleged that certain physicians connived to declare persons insane in order to obtain the fees. An investigation is being made.

---

IT has been claimed that one-fourth of the young men who have applied for admission to West Point have been unable to meet the physical qualifications.

---

THE second annual meeting of the Roentgen Society of the United States will be held at Buffalo on September 10 and 11, 1901, in the University building.

---

IMMIGRANTS suffering from consumption will be barred from entrance into Canada along similar lines as those inaugurated by the United States Immigration Department.

---

THE cutlery used for insane persons in England comprises a dull, round-bladed knife with a very short cutting edge, and a fork terminating in a ball, on which are three prongs about an inch long.

DR. J. P. KENNEDY has been elected Health Officer of Atlanta. Dr. Kennedy is an experienced sanitarian and will make a faithful and efficient officer.

---

DR. M. W. BARBOUR, of Arcola, Ill., treated a race horse and brought suit for his services. Judge Cochran ruled that a surgeon has no right to practice as a veterinarian.

---

A RECENT cable despatch says that the sultan of Turkey intends to present to the Berlin Hospital a wing, the plans of which have been sent to Emperor William for approval.

---

OWING to an epidemic of suicides in Emporia, Kansas, the mayor and Board of Health have forbidden the publication of details of suicides or attempts in local papers.

---

A "MAGNETIC HEALER" of Portland, Maine, was found guilty of practicing without a license. He added S. R. S. to his name, which he said meant "Silent Rubbing Specialist."

---

PERSONS having religious scruples against vaccination are exempt from the compulsory law of Wisconsin. How about those who have religious scruples against paying their debts?

---

THE College of Physicians and Surgeons, Chicago, has bought the West Division High School building for $186,000, and will also spend $40,000 on its building recently burned.

---

DR. T. V. HUBBARD, as the result of a street-car accident, sustained a dislocation of the elbow and fracture of the radius. Dr. Hubbard is in the hands of his professional friends, and is assuming the rôle of a Daniel come to judgment.

Dr. Remlinger, a graduate of the University of Lyons, and formerly a medical officer of the French army, has been appointed director of the Antirabic Institution of Constantinople.

---

Bernard M. Baker, of Baltimore, has presented the hospital ship, Maine, to the British government. This ship was originally fitted out for service in South Africa by American women.

---

A young Italian lady, Dr. Rina Monti, who has published several scientific papers and who gained a university gold medal, has been accepted by the University of Pavia as a lecturer in anatomy.

---

The Marine Hospital service will fight mosquitoes in the vicinity of its stations with kerosene. In New Jersey a Mosquito Exterminating Company has been formed. It will probably be a trust.

---

McGill University has been chosen as one of the institutions which will carry on original research work under the supervision of the newly incorporated Rockefeller Institute of Medical Research.

---

A negro woman in Decatur, Ala., gave birth to a child having only stumps for arms and legs. During pregnancy she witnessed the amputation of the limbs of a child that had been crushed by a locomotive.

---

Dr. J. W. Crump, of Steel's Depot, Ala., died at that place on April 25th, aged fifty-five years. Dr. Crump was an old and valued friend of this journal. He had been a subscriber for nearly a quarter of a century and had kept a complete file of The Journal since 1878. Dr. Crump was a well-known and useful practitioner and his loss will be felt. The sympathies of The Journal-Record are extended to his widow in her bereavement.

THE New York State board of health will take a census of the tuberculous in that State, through the physicians. The inquiry will have to do with the extent of tuberculosis, the effect of climate, and locations affected.

---

THE birth of the new Italian princess has been made the occasion for a handsome donation by the king of 200,000 lire ($40,000) to the Commune of Rome toward the erection of a sanitarium for tuberculous children.

---

DR. W. D. MADDUX died at his home at Monticello, Ga., on July 22. He was one of the charter members of the Georgia Medical Association and was said to have been the oldest practitioner in the State. He was born in 1814.

---

CHICAGO thinks of flushing the streets to keep them clean, while Milwaukee is about to abandon the method for a dry-sweeping patent process which draws the dust and dirt by an air blast into a chamber, so that it is not blown or scattered about.

---

THE annual address in medicine at Yale University was delivered by Professor Edmund B. Wilson, Ph.D., of Columbia University, on "The Higher Claims of Minute Research in Biology and Medicine," in the College Street Hall, Yale University, on Tuesday, June 25, at noon.

---

ACCORDING to a press despatch from New York, a census of consumptives is to be begun. It will be the first of the kind ever undertaken by that State. It is expected that it will throw light on the question of what the State should do for the care of those afflicted and who cannot afford to pay for treatment.

A METHOD for detecting human blood has been suggested by M. S. Cotton in the Bull. Soc. Chimique de Paris. It depends upon the fact that blood will liberate oxygen from hydrogen peroxid. Using 1 c.c. of blood with 250 c.c. of hy. per., he obtained for man, 580 to 610 c.c.: for horse and pig, from 320 to 350 c.c.; for ox, 165 to 170; for guinea-pig, 115 to 125, and for sheep, from 60 to 65 c.c. This large excess in man over all the lower species would seem to be of diagnostic value.

---

THE Army Board which has been in session in Washington for the examination of candidates for appointment in the medical corps of the army held its final meeting during the week of June 17, and then adjourned until next September. The results of the board's work have been, according to an exchange, disappointing in the few candidates who have met all the requirements. Since February not more than forty young physicians have proved their ability to receive commissions as assistant surgeons, and some of these were acting assistant surgeons in the service in the Philippines. The report of the board at Manila has been received at the War Department, and after all the appointments have been made there will still remain eighty vacancies in the one hundred and twenty-nine new positions, which are created by the army law of February 2.

